# Differences in Preferred Retinal Loci of Fixation in Binocular and Monocular Vision

**DOI:** 10.1167/iovs.67.11.44

**Published:** 2026-09-23

**Authors:** Maximilian Freiberg, Aleksandr Gutnikov, Christian Meltendorf, Stephan Reiss, Ralph Krüger, Wolf Harmening

**Affiliations:** 1 Department of Optometry, Berlin University of Applied Sciences and Technology, Berlin, Germany; 2 Department of Ophthalmology, University of Bonn, Bonn, Germany

**Keywords:** fixational stability, binocular scanning laser ophthalmoscopy, foveola, vergence disparity, binocular advantage

## Abstract

**Purpose:**

Fixational eye movements (FEMs) are critical for visual perception, yet their precise coordination during binocular viewing remains poorly understood at the microscopic retinal level. This study aimed to quantify differences in fixation stability and the spatial location of the preferred retinal locus (PRL) between monocular and binocular viewing conditions.

**Methods:**

Using a custom binocular scanning laser ophthalmoscope, we recorded high-resolution FEM sequences in 10 healthy subjects under monocular and binocular viewing. Fixation stability was evaluated using the non-parametric isoline contour area (ISOA). A permutation-based statistical framework was applied to account for the strong sample-to-sample correlation in high-speed eye-tracking data.

**Results:**

Results show that binocular viewing yields significantly smaller ISOA values compared to monocular viewing (*P* < 0.001), indicating a binocular advantage in fixation stability. This advantage is driven by a concurrent tightening of both components of fixational eye movements: smaller microsaccade amplitudes (median, 10.04 arcmin vs. 8.11 arcmin; *P* = 0.0098) and less dispersive drift (*P* ≤ 0.0039 across time lags of 33–833 ms). Furthermore, we identified systematic, yet individual, intra-subject spatial shifts of the PRL (∆PRL mean, 3.19 ± 2.20 arcmin) upon binocular viewing, which were predominantly driven by a disconjugate micro-vergence.

**Conclusions:**

Binocular viewing not only enhances oculomotor stability through smaller microsaccades and tighter drift but also actively shifts fixation to a different retinal location compared to monocular viewing. The visual system appears to tolerate objective vergence errors of several arcminutes to enable solving the binocular correspondence problem.

Even during fixation, the eyes are never perfectly stationary. By continuously shifting and drifting the retinal image across the underlying photoreceptor mosaic, fixational eye movements (FEMs) fundamentally shape how spatial visual information is encoded and processed.^1^ Rather than being random oculomotor noise, these miniature movements, specifically ocular drift, facilitate active retinal sampling, enabling visual resolution that can exceed the static sampling limits of the photoreceptor mosaic.^2^^,^^3^ In this context, the preferred retinal locus (PRL) acts as the functional anchor toward which the visual system actively relocates the target to maximize spatial information^4^^,^^5^ at the center of gaze. Yet, this understanding rests almost entirely on monocular measurements, whereas natural vision is binocular and requires the two eyes to be coordinated with arcminute precision.

Although the monocular PRL is known to be systematically offset from the anatomical cone density centroid (CDC) and remains stable over time,^6^^,^^7^ its behavior during the transition to binocular viewing is less clear. At a macroscopic scale, binocular viewing has been reported to improve fixation stability relative to monocular viewing in normally sighted observers.^8^^,^^9^ Two questions follow from this. First, if fixation is more stable under binocular viewing, which fixational eye movements underlie that gain, the fast microsaccades or the slow drift? Second, does binocularity merely stabilize the existing monocular anchor, or does it relocate the PRL to a different retinal position? Answering either question requires resolving movements on the arcminute scale,^10^^,^^11^ a regime that the macroscopic methods used in earlier work do not reach. How the binocular advantage is expressed at the level of the individual photoreceptor has therefore remained inaccessible.

Investigating this transition at the microscopic level requires specialized methodology. Historically, established technologies such as video-based eye trackers, scleral search coils, and dual Purkinje image (DPI) systems have pioneered the field, laying the groundwork for our current understanding of FEMs. Although these systems continue to offer distinct advantages depending on the research question, capturing absolute retinal coordinates poses specific challenges. Standard video-based eye trackers are widely applicable and non-invasive, but they often lack the spatial resolution required to reliably isolate miniature eye movements^12^^,^^13^ or can be influenced by pupil dynamics.^14^^,^^15^ High-resolution techniques, such as scleral search coils and DPI trackers, provide excellent spatiotemporal precision but come with specific trade-offs. Scleral search coils are limited by their invasiveness,^16^^,^^17^ whereas DPI trackers are susceptible to independent translations of the crystalline lens (lens wobble).^18^^–^^20^ Furthermore, conventional non-invasive systems typically infer gaze from anterior eye segment reflections and rely on subjective calibration protocols to map gaze direction to external screen coordinates. This indirect mapping can introduce systematic offsets when determining the absolute spatial location of a stimulus on the retina. Finally, although existing scanning laser ophthalmoscopy (SLO)-based binocular trackers circumvent anterior segment artifacts, they utilize dual-imaging systems that require fine temporal synchronization.^21^

To achieve unambiguous, calibration-free retinal tracking, we utilized a split-field binocular scanning laser ophthalmoscope (bSLO) that has been described previously.^22^^,^^23^ By employing a single light source to simultaneously image both eyes, our system provides intrinsically synchronized recordings of both retinae. A key feature of this system is the integration of an acousto-optic modulator (AOM), which acts as an optical switch to write visual stimuli directly into the scanning raster.^24^ Because the stimulus decrement is encoded directly into the resulting retinal video, this approach bypasses subjective calibration entirely, yielding an exact record of the trajectory of the target across the photoreceptor mosaic. We leveraged this setup to address the two questions raised above. We quantified how binocular viewing alters fixation stability (ISOA) and its underlying microsaccade and drift dynamics and how it relocates the preferred retinal locus (∆PRL), decomposing this shift into its version and vergence components, thereby testing the hypothesis that binocularity reorganizes the oculomotor strategy during fixation. As an exploratory analysis, we further asked whether the magnitude of the binocular shift relates to sighting eye dominance.

## Methods

### Subjects

A total of 10 healthy subjects (five male, five female; mean age, 33 ± 9 years) participated in the study. All participants were free of known ocular pathologies, possessed a decimal visual acuity of at least 1.0 in both eyes with best spherical correction, and reported no history of binocular vision disorders. Because the integrated Badal optometers solely compensate for spherical ametropia, individuals with a cylindrical refractive error (astigmatism) of ≥1.0 diopter (D) were excluded to ensure optimal image quality for both the bSLO recordings and the subject's visual percept. The study was conducted in accordance with the tenets of the Declaration of Helsinki and was approved by the Ethics Committee of the Medical Faculty, University of Bonn. Informed written consent was obtained from all participants prior to testing. To best preserve natural binocular viewing conditions and accommodate physiological pupil dynamics, measurements were performed under natural pupil conditions without pharmacological mydriasis.

### Apparatus and Stimulus Delivery

**Figure 1.**
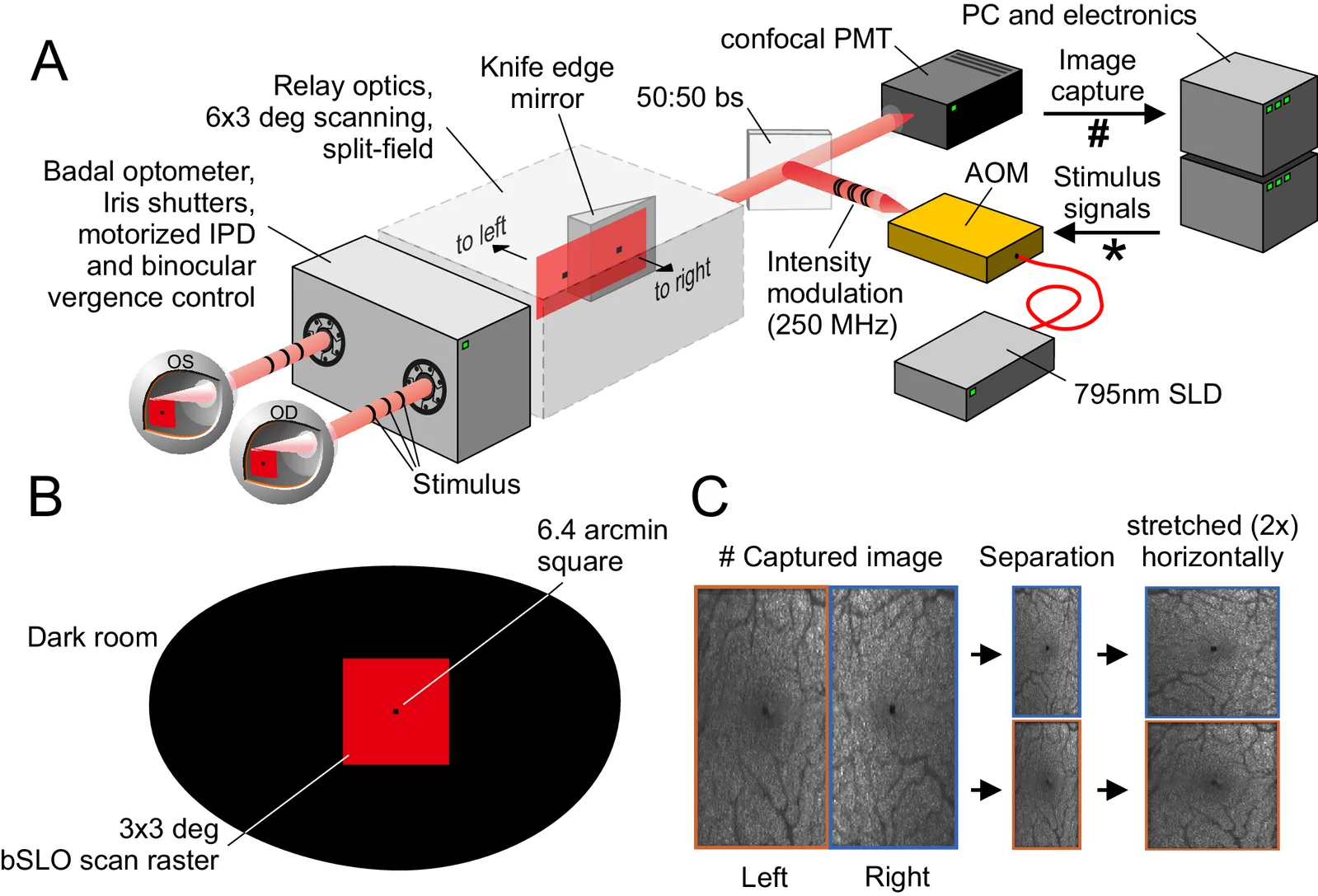
**Schematic representation of the bSLO setup and image processing.** (**A**) Optical setup: Path of the imaging beam from the 795-nm SLD through the AOM and scanner unit. The beam is separated by a knife-edge prism and guided through independent Badal optometers to correct for spherical ametropia. (**B**) Subject percept: The subject perceives, depending on the imaging condition, a monocular or binocularly combined 3° × 3° background raster. The fixation target is perceived in the center of this field, where the AOM has modulated the imaging beam. (**C**) Image acquisition and processing pipeline: The raw bSLO output is captured as a combined 512 × 512-pixel frame containing both the left-eye view (*orange border*) and the right-eye view (*blue border*), each compressed to 256 × 512 pixels (*left*). These interlaced images are subsequently separated into individual frames (*middle*) and digitally stretched by a factor of two along the horizontal axis to restore anatomically correct 512 × 512-pixel proportions (*right*). This reconstruction yields an undistorted view of the central 3° × 3° of the retina for both eyes.

FEMs were recorded using a custom-built, split-field bSLO (Fig. 1). The optical layout (Fig. 1A) utilized a single 795-nm superluminescent diode (SLD; SLD-CS-381-HP3-SM-795-I; Superlum, County Cork, Ireland) as a common light source. The beam was deflected by a scanner pair to generate a combined scanning raster of approximately 6° × 3° visual angle. A knife-edge prism split this beam into two equal 3° × 3° subfields for the left and right eye, respectively. The optical path of each eye was equipped with an independent Badal optometer to compensate for individual spherical refractive errors (ranging from approximately +2 to −7 D).^23^ Furthermore, each optical path featured an automated iris shutter, enabling the independent and instantaneous physical occlusion of either imaging channel. Finally, the light backscattered from both retinae was recombined at the prism and detected by a shared photomultiplier tube (PMT; H7422-50; Hamamatsu Photonics, Hamamatsu City, Japan), ensuring intrinsic temporal synchronization between the two optical paths. PMT signals were sampled by a field programmable gate array to produce 512 × 512-pixel video frames at 30 Hz, producing a digital resolution of 168 pixels per degree of visual angle.

This configuration fixed the vergence and accommodative demands at zero. Because the two beam paths emerged strictly parallel from the system and the fixation target was written to the same, corresponding raster position in both subfields, the stimulus was presented at optical infinity with zero lateral offset between the eyes; the vergence demand was therefore zero (*V*_0_ = 0), identical in the monocular and binocular conditions. Inter-pupillary distance was not recorded; because the beam paths remained parallel regardless of their lateral separation, adjusting for inter-pupillary distance translated the beams to each subject's eyes without introducing a vergence demand. Likewise, because the Badal optometers imaged the stimulus to a sharp retinal focus at optical infinity, the accommodative demand was approximately zero and identical across conditions; only the spherical refractive error was corrected, and no additional defocus was introduced.

The fixation target was a black square with a retinal extent of 6.4 × 6.4 arcmin. This target was generated by an AOM (Brimrose Corporation, Sparks, MD, USA), which acts as a high-speed optical switch within the imaging beam. By selectively switching off the SLD at the precise coordinates within the scanning raster, the AOM writes the stimulus directly into the retinal image (Fig. 1B). Although a wavelength of 795 nm lies in the near-infrared spectrum, the focused intensity of the SLD, delivering approximately 65 µW of optical power to each eye, provides sufficient stimulation to the extreme long-wavelength tail of the L-cone sensitivity curve.^25^ Consequently, subjects perceive the scanning raster as a deep red background. During binocular viewing, the two 3° × 3° scanning subfields are combined into a single, shared background raster, within which the central dark fixation target is perceived.

### Experimental Procedure

While the head was stabilized in front of the bSLO via a dental impression (bite bar) on an XYZ-translation stage, participants were asked to fixate on the central target as relaxed and accurately as possible. The experiment followed a randomized block design comprising three viewing conditions: monocular right (OD), monocular left (OS), and binocular (both eyes open). To ensure strictly monocular viewing without residual light leakage, the optical path to the contralateral eye was physically blocked using the integrated automated iris shutter. In the binocular condition, both shutters were opened, presenting the target to both eyes simultaneously. For each condition, five separate 10-second video sequences were recorded, yielding a total of 15 videos per participant.

### Data Analysis

**Figure 2.**
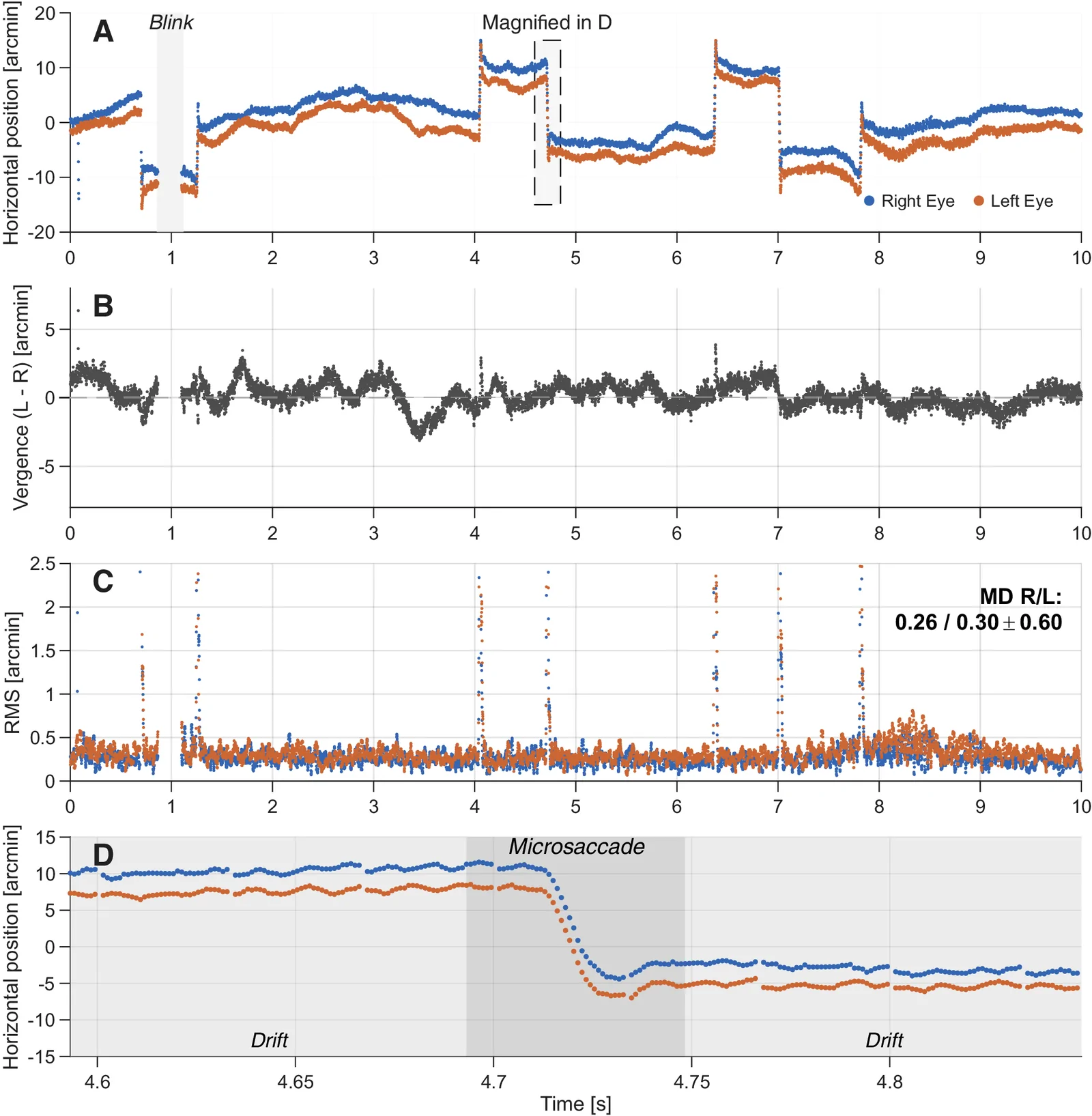
**Representative binocular eye trace and oculomotor event analysis.** (**A**) Horizontal position over a 10-second trial for the right eye (*blue*) and left eye (*orange*). Both traces share a common origin (mean of the medians of the two eyes), making their conjugacy visible; positive values are rightward. A blink (around second 1) is shaded *gray*; the *dashed box* is magnified in **D**. (**B**) Instantaneous horizontal vergence (L–R), with each eye referenced to its own monocular PRL; positive values correspond to rightward displacement of the left relative to the right eye (i.e., a more convergent [eso] posture). **A** and **B** use the reference suited to each: a shared origin for conjugate dynamics in **A**, and the monocular PRLs for relative alignment in **B**. (**C**) Moving root-mean-square deviation (20-ms window), reflecting the noise floor; median values for each eye are indicated. (**D**) High-zoom view of a binocular microsaccade, showing high conjugacy between fellow eyes. Shading indicates slow intersaccadic drift (*light gray*) and the rapid microsaccade (*dark gray*).

Because the bSLO constructs images over time, continuous fixational eye motion generates spatial distortions within each acquired video frame. To recover the underlying oculomotor trajectories, post-processing was performed offline in MATLAB (MathWorks, Natick, MA, USA) using a strip-wise image registration algorithm.^26^^,^^27^ Each 30-Hz video frame was segmented into 32 horizontal strips. By cross-correlating each strip against a reference frame generated from the respective video, its spatial offset was determined. This process “stabilizes” the video and deconvolves the eye motion from the raster scan, extracting the stimulus trajectory and yielding continuous two-dimensional motion trajectories with an upsampled temporal resolution of 960 Hz. A representative excerpt of these trajectories, highlighting fixational phenomena such as drift and microsaccades, is illustrated in Figure 2.

To identify tracking artifacts, a Hampel filter was employed for robust outlier detection. Data points deviating from the local median by more than 3 SD were classified as non-physiological noise spikes and removed. Imaging parameters, such as PMT gain, were individually adjusted to optimize image contrast. As an additional quality control step, we calculated the variance of the positional samples relative to a 10-sample moving average. Videos demonstrating excessive tracking noise (variance ≥ 3 pixels, equating to 1.07 arcmin) were excluded, and the five best-quality videos per condition and participant were retained for analysis.

To establish a consistent spatial coordinate system across recordings, the exact center of the stimulus square was identified in the first frame of every video. This center point was defined as the origin (0, 0). To aggregate the discrete fixation trials into a single point cloud for each condition, all individual reference frames were aligned to a single master reference image using the MATLAB function **imregcorr**. Mapping all coordinates onto this unified retinal coordinate system occasionally required manual refinement using distinct retinal landmarks, such as blood vessels.

### Fixation Metrics and Statistical Framework

Fixational behavior was quantified using two spatial metrics:

1. PRL: the spatial median of the pooled *x*, *y* coordinates, used to account for non-Gaussian outliers. The individual functional fixation shift (∆PRL) was calculated as the absolute Euclidean distance between the monocular and binocular medians within each eye.
2. ISOA: the probability density area encompassing 68% of the highest-density fixation points, derived from a two-dimensional (2D) kernel density estimation and therefore avoiding the assumptions of bivariate normality. The ISOA was used as the measure of fixation stability.

**Figure 3.**
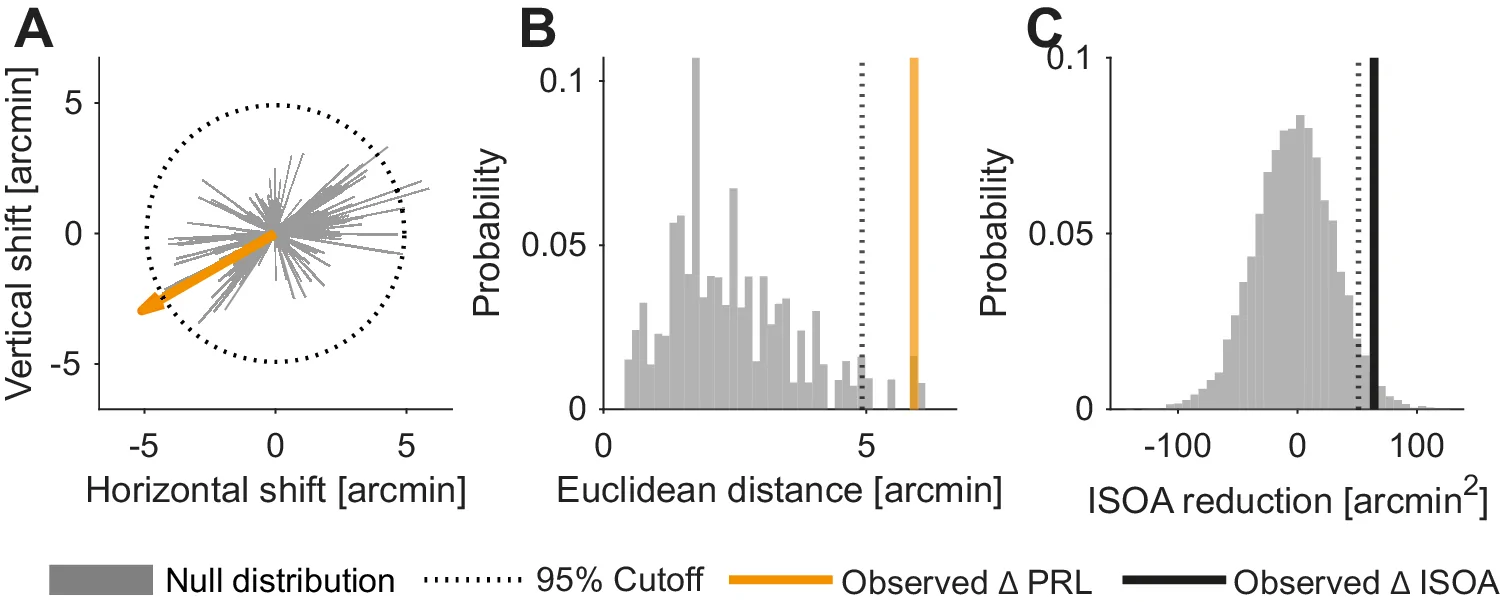
**Permutation-based statistical framework for individual spatial shifts and fixation stability.** Data are shown for a representative subject (BAK8060, left eye). Empirical null distributions were generated using a block-permutation approach (10,000 iterations) that preserved the sample-to-sample correlation of the oculomotor signal by permuting whole blocks, 1-second epochs for the ISOA analysis, and whole trials for the ∆PRL analysis. (**A**) Two-dimensional null distribution of pseudo-shift vectors (*gray cloud*). The *gold arrow* indicates the observed spatial shift (∆PRL). The *dotted black circle* represents the 95% significance threshold. (**B**) The corresponding one-dimensional null distribution of shift magnitudes. The observed ∆PRL (*gold line*, 5.9 arcmin) falls to the right of the 95% cutoff (*dotted line*, 4.6 arcmin), indicating a significant spatial shift (*P* < 0.05). (**C**) One-dimensional empirical null distribution for the difference in fixation stability (∆ISOA). The observed reduction in area (*black line*, 64 arcmin^2^) exceeds the 95% cutoff (*dotted line*, 55 arcmin^2^), indicating a significant difference in fixation stability (*P* < 0.05).

Because high-frequency eye-tracking data exhibit strong sample-to-sample correlation, successive samples are not statistically independent, and standard parametric tests, which assume independence, can lead to pseudoreplication and an inflated false-positive rate. To avoid this, we assessed significance with a non-parametric block-permutation test.^28^ For each analysis, the recording was divided into contiguous blocks whose length preserved the sample-to-sample correlation of the signal: 1-second epochs for the ISOA comparison and whole trials for the ∆PRL comparison. The monocular and binocular blocks were then pooled and randomly reassigned to the two conditions to build an empirical null distribution, against which the observed ∆PRL and ISOA differences were tested. Permuting whole blocks rather than individual samples preserved the within-block temporal structure and thus respected the non-independence of the data (Fig. 3).

### Statistical Analysis of Spatial Fixation Shifts (∆PRL)

To assess the specific spatial fixation shifts (∆PRL) within individual eyes, permutations were performed strictly at the trial level to preserve the biological variance of the initial targeting strategy of the oculomotor system. Condition labels (monocular vs. binocular) were randomly shuffled across intact trials rather than individual gaze samples. The Euclidean distance between the spatial medians of these pseudo-conditions was then calculated to build the discrete null distribution (Fig. 3B). Comparing the observed ∆PRL against this distribution provided a robust evaluation of spatial relocation. Unless otherwise stated, all descriptive group-level statistics and variance measures are reported as mean ± SD. Paired group-level comparisons between viewing conditions were assessed with two-sided Wilcoxon signed-rank tests.

### Microsaccades, Drift, and Ocular Dominance

To resolve the fixational behavior into its constituent kinematic components, we characterized both the rapid microsaccadic and the slow, intersaccadic drift episodes of the oculomotor signal. Microsaccades were detected on the 960-Hz trajectories using the velocity-based algorithm of Engbert and Kliegl,^29^ with a median-based velocity threshold of λ = 5 applied as a 2D elliptic criterion, a minimum duration of 8 ms, and a minimum intersaccadic interval of 20 ms; velocity was computed over a five-sample moving window. Detected events were subsequently verified by an experienced grader (one of the authors) to reject residual tracking artifacts. For each eye, we quantified microsaccade rate, defined as the number of events per second of valid fixation, and microsaccade amplitude in arcmin. To isolate the intersaccadic drift, microsaccades (extended by a 20-ms postsaccadic buffer), blinks, and invalid registrations were removed from the trace. The remaining trace was segmented into continuous drift episodes (minimum duration 50 ms), bridging registration gaps shorter than one video frame (33 ms) so that transient tracking dropouts did not spuriously truncate an otherwise continuous drift. The residual dispersion of this drift was quantified via the mean squared displacement (MSD). Drift trajectories **r**(*t*) = [*x*(*t*), *y*(*t*)] (in arcmin) were obtained at video-frame resolution by averaging all strips within each frame, and the MSD was computed over all point pairs separated by a fixed lag, τ, pooled within each participant and condition:

$$\mathrm{MSD}\left(\tau \right)={\langle \;∥r\left(t+\tau \right)-r\left(t\right){∥}^{2}\;\rangle }_{t}$$

where ∥·∥ is the Euclidean norm, and point pairs were formed only within contiguous drift segments (not across blinks or removed microsaccades). Lags (τ) were converted to milliseconds using the video frame rate.

To examine whether the observed spatial shifts were governed by ocular dominance, sighting dominance was determined for each participant using the Miles test, and the per-eye ∆PRL values were regrouped into dominant and non-dominant eyes for a paired comparison. One participant showed indeterminate dominance and was excluded from this comparison, leaving a predominantly right-dominant cohort (six right-dominant, three left-dominant).

## Results

### The Binocular Advantage in Fixation Stability

**Figure 4.**
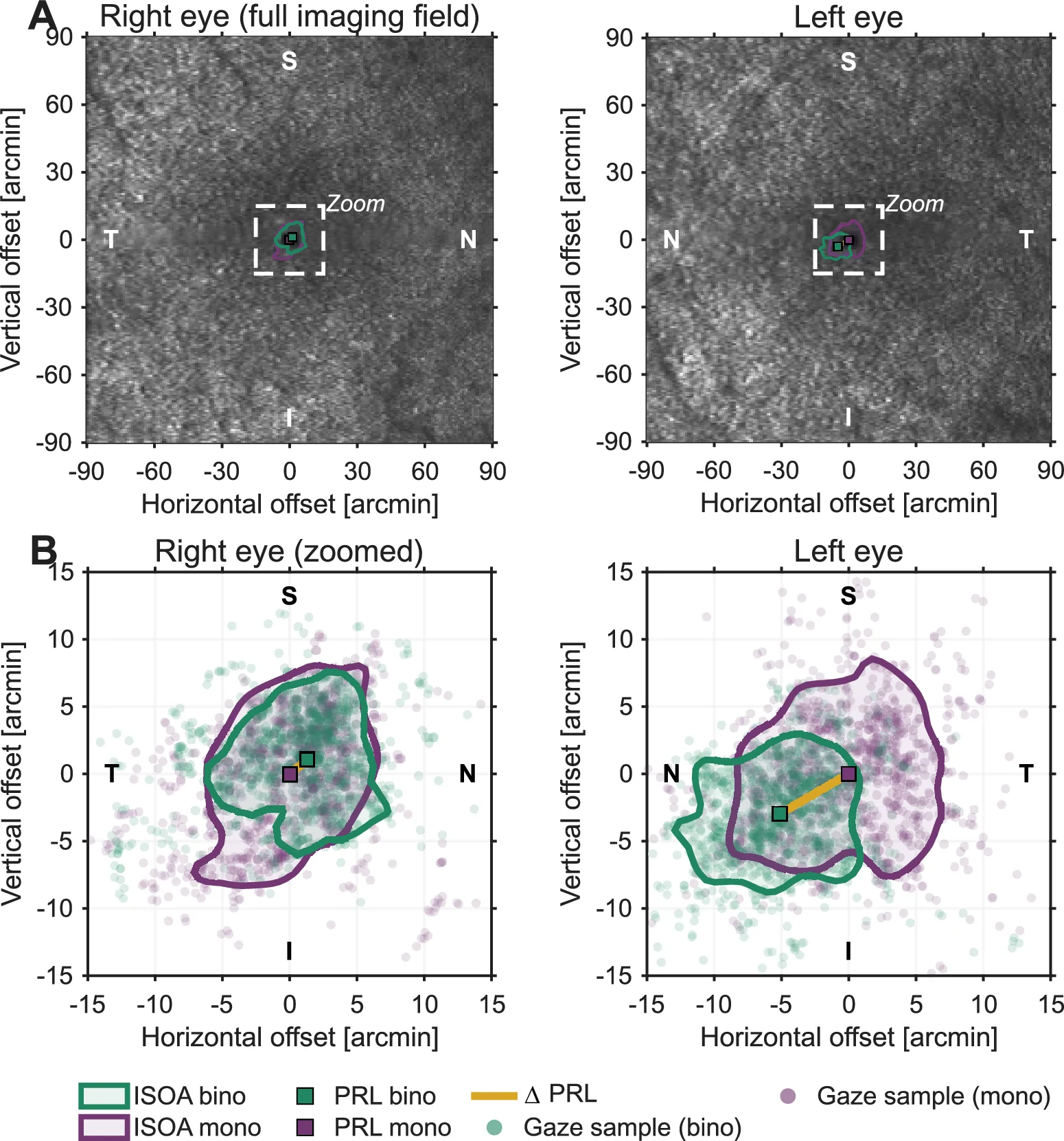
**Spatial distribution of fixational gaze and PRL.** (**A**) Fixation locations overlaid on the corresponding bSLO images of the central retina (3° × 3° imaging field). The *dashed white square* delineates the region of interest magnified below. (**B**) Magnified view (±15 arcmin) of the central fixation areas, visualizing individual gaze samples (semitransparent dots) without the SLO background. Data are shown for monocular (*plum*) and binocular (*emerald green*) viewing conditions. *Solid squares* denote the spatial median (PRL). Contour lines enclose the 68% ISOA. The *gold vector* represents the shift (∆PRL) between monocular and binocular PRLs. Anatomical orientations on the retina: superior (S), inferior (I), temporal (T), and nasal (N). Data are shown for a single representative subject (BAK8060), with all positions given in retinal coordinates.

To determine whether binocular viewing enhances oculomotor stability, we compared the ISOAs between viewing conditions. At the individual level, transitioning to binocular viewing generally resulted in a tighter spatial clustering of fixation points (Fig. 4). By applying the trial-wise permutation framework to the ISOA metric, we confirmed that this intra-individual gain in stability was statistically significant (*P* < 0.05) for at least one eye in eight out of 10 subjects. The validity of this intra-individual approach is illustrated by the empirical null distribution of a representative subject in Figure 3C.

**Figure 5.**
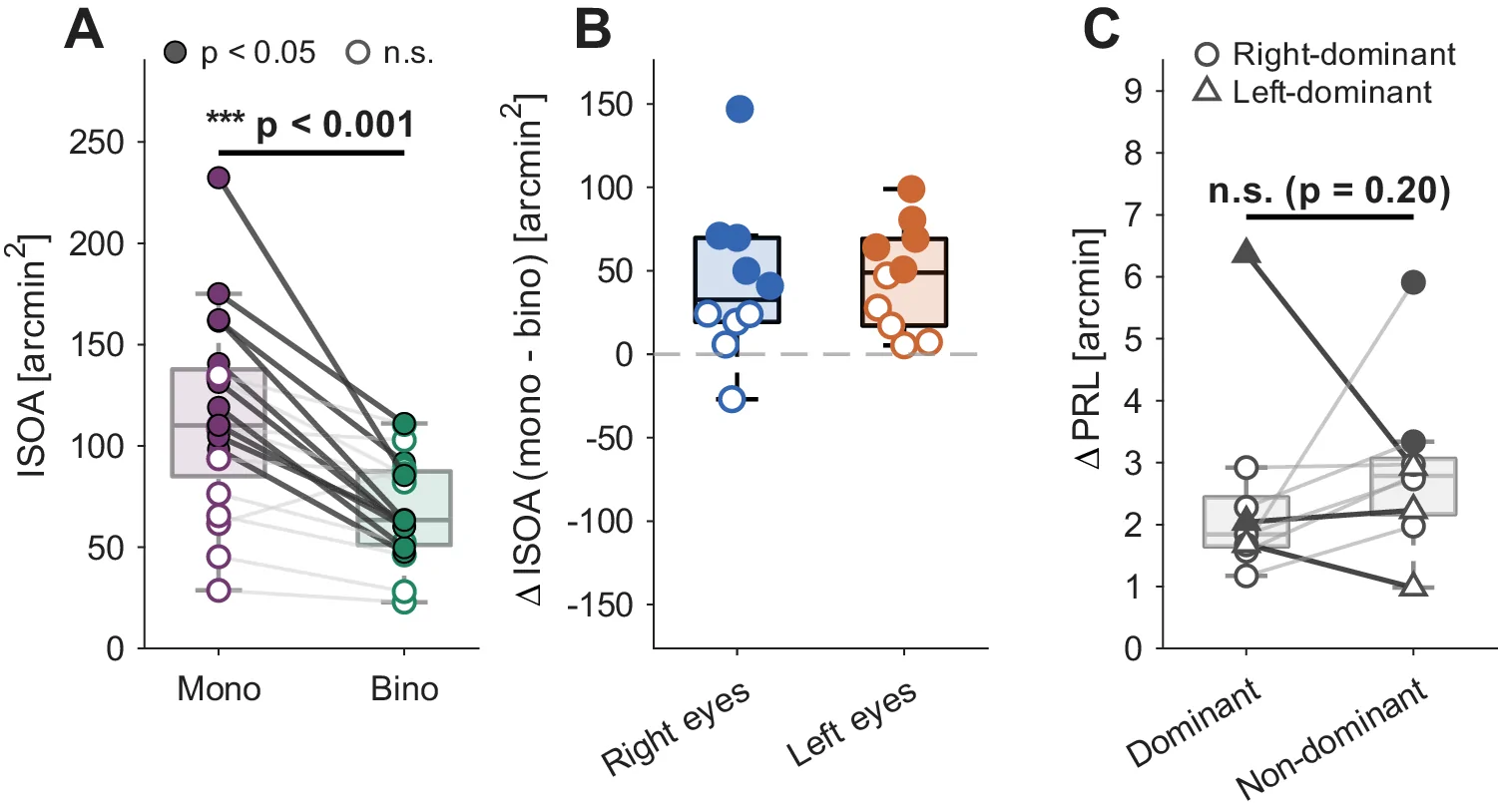
**Fixation stability and magnitude of intraocular PRL shifts.** (**A**) Paired slopegraph of absolute ISOA, monocular versus binocular; *lines* connect individual eyes, and *boxplots* show the group distribution. Each condition comprises 20 data points (10 participants × 2 eyes). Binocular viewing significantly reduced ISOA (*P* < 0.001, Wilcoxon signed-rank test). (**B**) ∆ISOA (ISOA_mono_ – ISOA_bino_), by right and left eyes; *positive values* indicate improved binocular stability. *Filled markers* denote individually significant differences in stability (*P* < 0.05, permutation test), and *open markers* indicate non-significant ones. (**C**) Magnitude of the PRL shift (∆PRL) between conditions, grouped by sighting dominance (*n* = 9; one participant with indeterminate dominance excluded); left-dominant participants are emphasized (*dark lines*). *Filled* and *open markers* are as in **B**, here denoting individually significant versus non-significant shifts. The dominant eye did not shift significantly less than the non-dominant eye (median, 1.84 arcmin vs. 2.79 arcmin; *P* = 0.20, paired Wilcoxon signed-rank test). A leave-one-out analysis indicated this null result depends on a single participant (see Results).

At the group level, this binocular advantage in control was highly consistent across the cohort (Fig. 5A). Under purely monocular viewing, the average ISOA was 107.43 ± 58.49 arcmin^2^ in right eyes and 114.52 ± 37.09 arcmin^2^ in left eyes. During binocular viewing, the mean ISOA was reduced to 62.01 ± 28.83 arcmin^2^ (OD) and 72.72 ± 26.27 arcmin^2^ (OS). A Wilcoxon signed-rank test confirmed that this systematic reduction in fixational spread was statistically significant across participants (*P* < 0.001) (Fig. 5A).

### Microsaccadic and Drift Contributions to the Binocular Advantage

**Figure 6.**
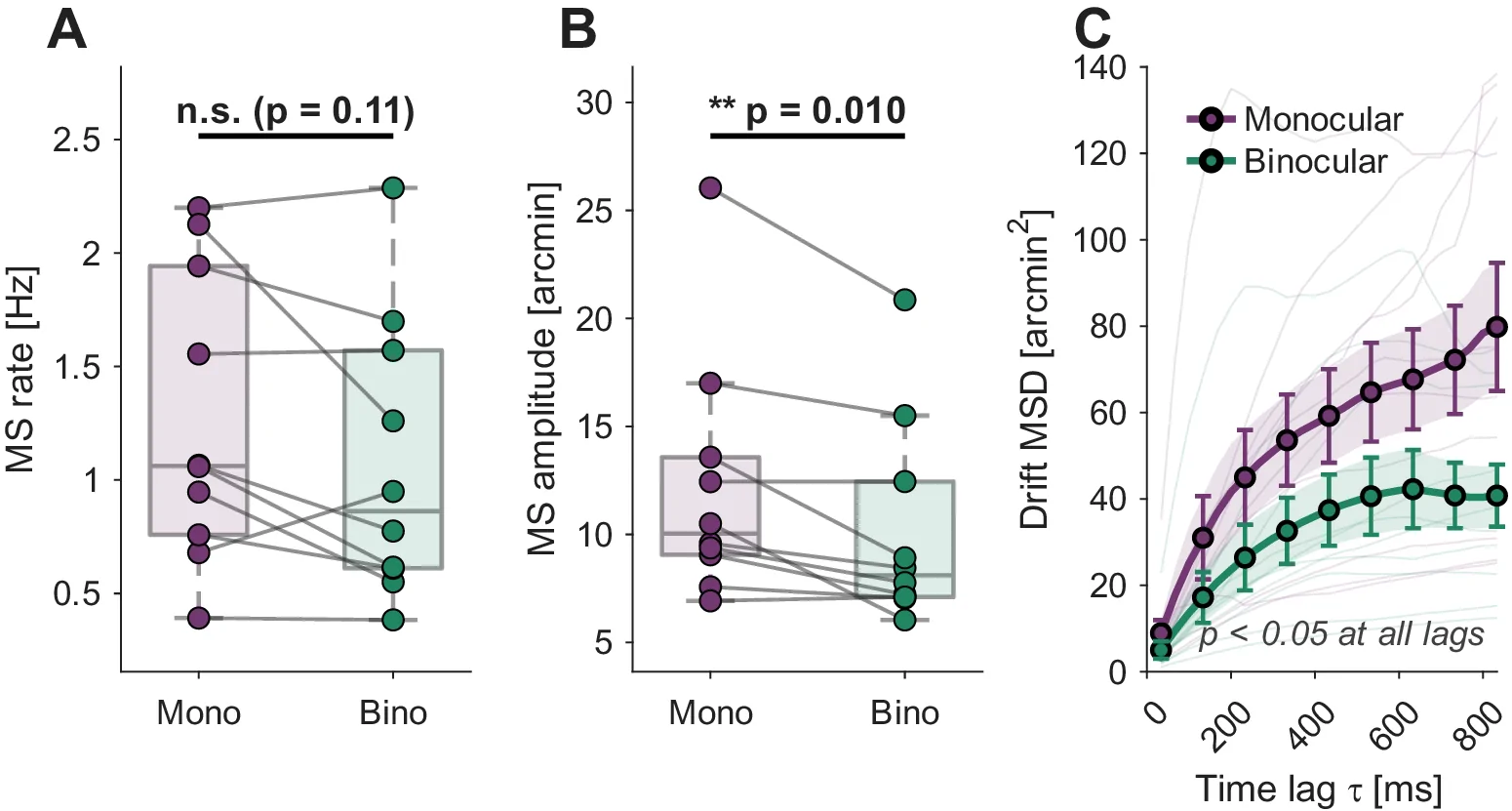
**Fixational eye movements under monocular and binocular viewing** (*n* = 10; per-participant medians, paired Wilcoxon signed-rank tests). (**A**) Microsaccade rates did not differ between conditions (1.06 Hz vs. 0.86 Hz; *P* = 0.11). (**B**) Microsaccade amplitude was smaller binocularly (10.04  arcmin vs. 8.11 arcmin; *P* = 0.0098). (**C**) MSD of ocular drift versus time lag τ (video-frame trajectories, microsaccades removed). Binocular drift dispersed less at every lag from 33 to 833 ms (all *P* ≤ 0.0039; at τ = 600 ms, 66.85 arcmin^2^ vs. 41.98 arcmin^2^). *Lines* show the group mean; *bands* and *error bars* ± 1 SEM.

Having established a global gain in fixational stability, we sought to determine which kinematic component of the oculomotor signal underlies this binocular advantage by comparing microsaccades and ocular drift across viewing conditions (Fig. 6). The rate of microsaccades did not differ significantly between monocular and binocular viewing (median, 1.06 Hz vs. 0.86 Hz; *P* = 0.11) (Fig. 6A), indicating that the binocular advantage is not explained by a change in how often microsaccades occur. The amplitude of these microsaccades, however, was significantly reduced under binocular viewing (median, 10.04 arcmin vs. 8.11 arcmin; *P* = 0.0098) (Fig. 6B), so that microsaccades relocated gaze over shorter distances.

In parallel, the slow intersaccadic drift exhibited a comparable constriction. The mean squared displacement of the drift was consistently lower during binocular than during monocular fixation across every time lag examined, from 33 to 833 ms (all *P* ≤ 0.0039) (Fig. 6C). The magnitude of the effect was stable across the sampled interval, with monocular drift dispersing over roughly twice the area of its binocular counterpart; at a representative lag of τ = 600 ms, for example, the mean squared displacement was 66.85 arcmin^2^ versus 41.98 arcmin^2^ (*P* = 0.0039), with binocular drift smaller in nine of 10 participants. Taken together, the concurrent reduction in microsaccade amplitude and drift dispersion demonstrates that binocular viewing tightens both constituent components of fixational eye movements, providing a mechanistic substrate for the observed reduction in ISOA.

### Spatial Shift of the PRL

Beyond global stability, we investigated whether the absolute functional center of fixation (the PRL) shifted spatially upon transitioning from a monocular to a binocular state. We quantified this intraocular relocation as the absolute Euclidean distance (∆PRL) between the monocular and binocular spatial medians within a single eye. The magnitude of these spatial shifts ranged between 0.98 and 9.30 arcmin across subjects, with a mean ∆PRL of 3.19 ± 2.20 arcmin. Importantly, to distinguish true functional relocation from inherent oculomotor noise, trial-wise permutation testing was applied to each individual eye. This analysis revealed that these spatial shifts were statistically significant (*P* < 0.05) for at least one eye in half of the participants (five out of 10).

**Figure 7.**
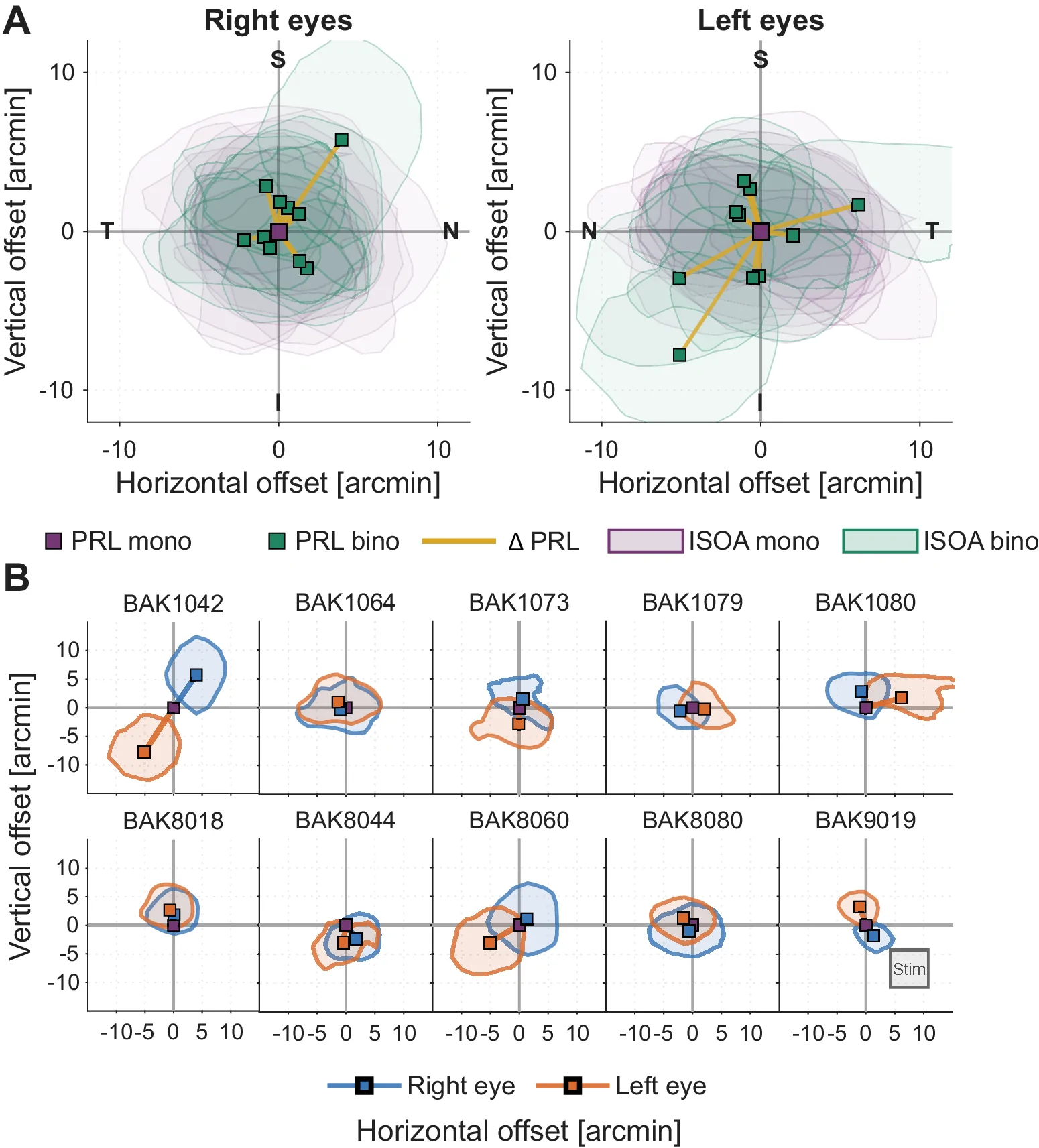
**Individual and group-level spatial distribution of binocular PRL shifts.** (**A**) Group data. Monocular PRLs are anchored at the origin (0, 0) to show relative binocular relocation (∆PRL, *gold vectors*). *Shaded regions* indicate 68% ISOAs for monocular (*plum*) and binocular (*emerald green*) conditions. Anatomical orientations: superior (S), inferior (I), temporal (T), and nasal (N). (**B**) Individual data. Binocular offsets for the right (*blue*) and left (*orange*) eyes are plotted relative to a shared monocular baseline (*plum square*). *Solid lines* show intraocular ∆PRL vectors. Although ∆PRL captures single-eye relocation, the interocular difference between the OD and OS vectors constitutes the objective fixation disparity relative to the zero vergence demand. *Shaded regions* denote binocular ISOAs. The *gray box* (‘Stim’) shows the 6.4 × 6.4-arcmin stimulus for visual reference.

Furthermore, mapping the two-dimensional spatial distribution of these intraocular shifts for the entire cohort (Fig. 7A) and at the individual level (Fig. 7B) revealed high inter-individual variability in both shift magnitude and absolute spatial direction. The precise 2D retinal coordinates recruited for binocular viewing varied considerably across the cohort, indicating that the new functional anchor is not simply determined by a rigid, universally shared anatomical landmark. Within this distribution, the left eyes tended to exhibit both a larger median shift (2.88 arcmin vs. 2.04 arcmin) and a wider spread of ∆PRL values across subjects than the right eyes (interquartile range [IQR], 3.87 arcmin vs. 1.35 arcmin), visible as the broader scatter of the left-eye vectors in Figure 7A.

Given the pronounced inter-individual variability in both the magnitude and direction of these shifts, we next asked, in an exploratory analysis, whether this heterogeneity was structured by ocular sighting dominance, reasoning that a dominant eye might relocate less upon binocular viewing (Fig. 5C). The dominant eye exhibited a numerically smaller shift in seven of nine participants (one participant with indeterminate dominance was excluded), a trend in the expected direction that did not reach statistical significance (median, 1.84 arcmin vs. 2.79 arcmin; *P* = 0.20, paired Wilcoxon signed-rank test). The present data therefore do not resolve whether sighting dominance structures the binocular relocation of the PRL. A leave-one-out sensitivity analysis showed that this null result was not robust: It hinged on a single participant, whose dominant eye shifted markedly more than the non-dominant eye (opposite to the group tendency). Excluding this participant rendered the difference significant (*P* = 0.039). We report this as a post hoc observation; the dominance effect thus remains suggestive but statistically fragile in this cohort.

### Kinematic Decomposition of PRL Shifts

**Figure 8.**
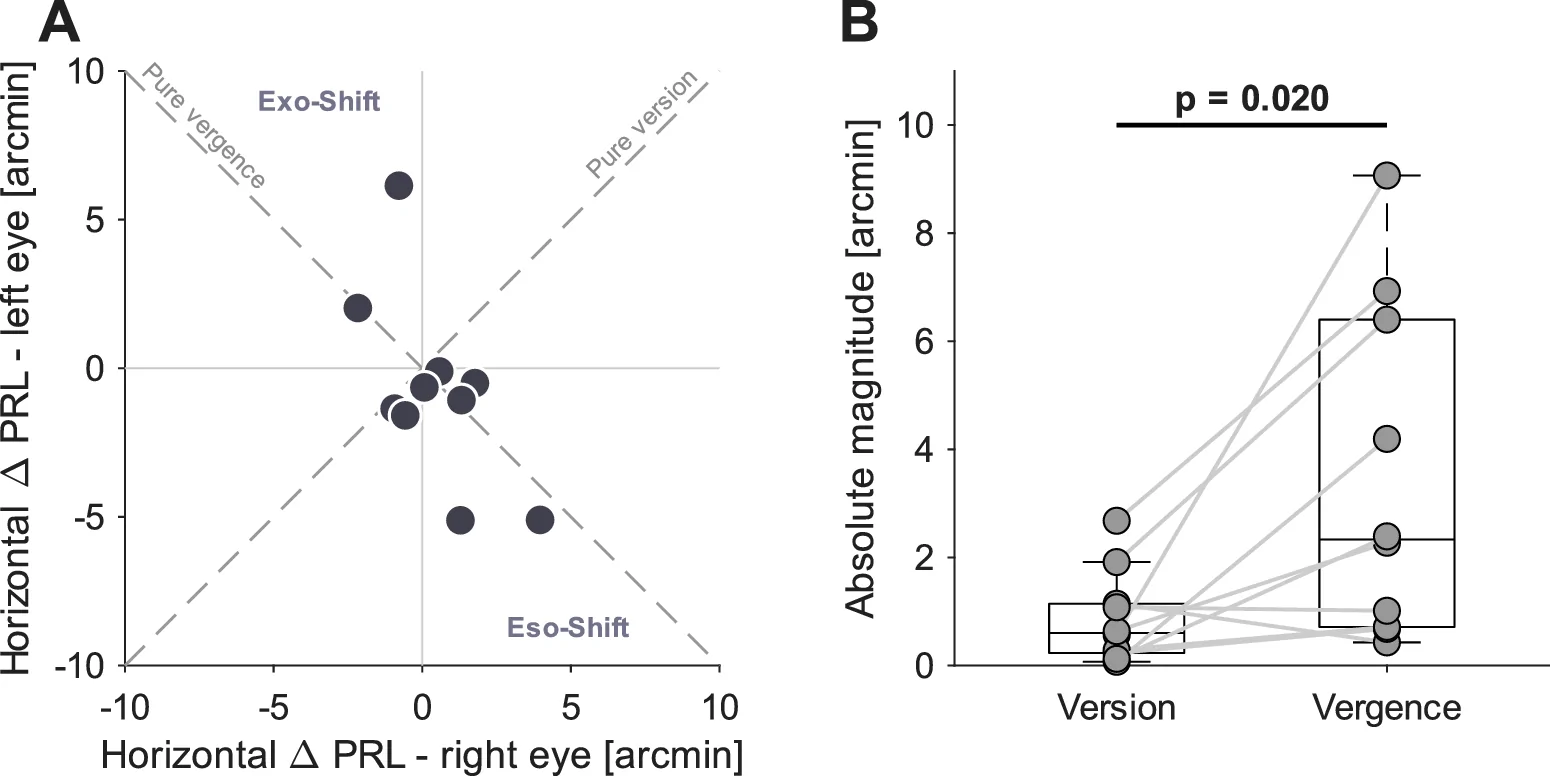
**Kinematic decomposition of binocular PRL shifts.** (**A**) Quadrant analysis of horizontal shift magnitudes (∆*X*) for the right and left eyes. *Dashed diagonals* mark the two limiting cases: Along the main diagonal, both eyes shift equally in the same direction (pure version), whereas along the counter-diagonal they shift equally in opposite directions (pure vergence). Most participants fall closer to the pure-vergence diagonal, in the upper-left (exo) or lower-right (eso) quadrant, indicating a predominantly disconjugate (micro-vergence) shift. (**B**) Mathematical decomposition of the horizontal shifts into their conjugate (version) and disconjugate (vergence) components. Individual lines represent paired data for each subject. The absolute magnitude of the micro-vergence component is significantly larger than the version component (*P* = 0.020, Wilcoxon signed-rank test), suggesting that the observed PRL relocation reflects a micro-vergence adjustment during the transition to binocular viewing.

To understand the binocular coordination driving these individual spatial shifts, we decomposed the horizontal relocation vectors into their conjugate (version) and disconjugate (vergence) components. Denoting the horizontal PRL shift of the right and left eye by ∆*X*_OD_ and ∆*X*_OS_, respectively, the version component was defined as their mean, version = (∆*X*_OD_ + ∆*X*_OS_)/2, and the vergence component as their difference, vergence = ∆*X*_OD_ − ∆*X*_OS_. With this convention, a positive right-eye shift and negative left-eye shift (∆*X*_OD_ > 0, ∆*X*_OS_ < 0) yield a positive vergence value and correspond to an eso-shift (over-convergence), whereas the opposite pattern corresponds to an exo-shift; the two occupy opposite disconjugate quadrants (Fig. 8A). A purely conjugate shift would indicate a parallel displacement of both eyes, whereas a disconjugate shift reflects a change in the vergence angle.

Plotting the horizontal shift of the right eye against the left eye (Fig. 8A) revealed a predominantly mirror-symmetric distribution. The majority of individual shift vectors fell into the disconjugate quadrants, with a notable predominance of binasal (eso) shift. To quantify this observation, we compared the absolute magnitudes of the version and vergence components (Fig. 8B). The magnitude of the micro-vergence shift was significantly larger than the version shift (*P* = 0.020, Wilcoxon signed-rank test). This demonstrates that the visual system does not simply undergo random spatial jitter upon binocular viewing but rather actively introduces a functional micro-vergence to establish binocular correspondence.

## Discussion

In this study, we investigated the oculomotor consequences of binocular viewing with spatial precision approaching the scale of individual photoreceptors. By comparing monocular viewing to a binocular state at a fixed focal depth, we observed two primary outcomes: an improvement in fixation stability and individual spatial shifts of the PRL. The use of the split-field bSLO architecture helps avoid the artifacts commonly associated with conventional trackers, such as pupil diameter fluctuations in video-based systems or post-saccadic crystalline lens oscillations in DPI trackers.^18^^–^^20^ By bypassing these anterior segment dynamics through direct retinal imaging, we can confidently conclude that the measured reductions in ISOAs and the observed spatial relocations reflect genuine neural and motor adaptations during binocular fixation.

### Binocular Optimization of Fixation Stability and Active Sampling

Our results show that binocular viewing reduces the spatial spread of FEMs compared to monocular viewing. This provides microscopic evidence for the binocular advantage reported in previous macroscopic studies.^8^^,^^9^^,^^30^ This tighter control might not simply represent a suppression of eye motion. Recent evidence suggests that physiological ocular drift acts as an active sampling mechanism.^2^^,^^3^ By constraining the global fixational spread during binocular viewing, the oculomotor system may optimize the spatiotemporal window for this active sampling. Our kinematic decomposition of the fixational signal indicates that this gain in stability is not attributable to a single dominant mechanism. Rather, binocular viewing was accompanied by a concurrent reduction in both microsaccade amplitude and drift dispersion (Fig. 6), suggesting that the binocular advantage arises from a coordinated tightening of both the slow and the rapid constituents of fixational eye movements. Because more confined ocular drift keeps the sampled image within the region of highest cone density and has been associated with better acuity in monocular studies,^2^^,^^31^ the reduced drift dispersion we observed under binocular viewing could contribute to the binocular acuity advantage frequently reported in the literature.

Crucially, this optimization aligns with the established psychophysical literature on binocular sensory advantages. It is well known that binocular viewing lowers contrast thresholds and improves visual acuity, a phenomenon broadly termed binocular summation.^32^^–^^34^ Although these sensory gains are traditionally attributed to neural convergence in the primary visual cortex, a necessity given that pre-cortical parvocellular pathways preserve single-cone resolution,^35^^,^^36^ our findings suggest a synergistic motor component. By physically constraining the fixational spread (reduced ISOA), the eyes maintain the target within a narrower, highly sensitive region of the foveola. This optimized alignment likely minimizes interocular decorrelation, thereby potentially providing the visual cortex with a more stable, congruent binocular signal that is beneficial for fine stereopsis (i.e., stereoacuity).^37^

### From Intraocular PRL Shifts to Objective Fixation Disparity

At the level of the individual eye, the PRL shifted by a Euclidean distance of up to 9.30 arcmin upon binocular viewing. This ∆PRL is an intraocular quantity: a two-dimensional vector describing the relocation of the PRL of a single eye from monocular to binocular viewing, with both horizontal and vertical components. Our kinematic decomposition (Fig. 8) links these single-eye shifts to binocular alignment. It reveals that the individual ∆PRLs are not merely parallel conjugate movements but rather reflect a predominantly mirror-symmetric, disconjugate micro-vergence. The objective fixation disparity (oFD) follows from the horizontal components of the shifts of the two eyes: It is the interocular difference between them, and thus characterizes the horizontal realignment of the binocular system as a whole rather than the relocation of either eye alone. Conventionally it is expressed as oFD = *V* − *V*_0_, where *V* is the measured vergence and *V*_0_ is the vergence demanded by the stimulus.^38^^–^^41^ Because our paradigm used parallel beam paths presenting the target at corresponding retinal positions, the vergence demand was strictly zero (*V*_0_ = 0; see Methods), so the disparity reduces to the measured horizontal vergence itself. Referenced to this fixed zero, its direction is unambiguous: Residual convergence corresponds to an eso-disparity (over-convergence) and residual divergence to an exo-disparity.

The objective fixation disparity considered here, the residual horizontal vergence offset present during steady binocular fixation, is distinct from heterophoria, the vergence deviation that emerges only when fusion is disrupted and the eyes are dissociated. The binocular PRL thus represents a functional position adopted during binocular viewing, associated with a disconjugate micro-vergence rather than a fixed anatomical target. Whether it bears a systematic relationship to the underlying retinal topography, as the monocular PRL does to the anatomical cone density centroid,^6^ remains an open question.

This observation aligns with, yet expands upon, recent findings by Bowers et al.^21^ Using a comparable binocular tracking SLO approach, they demonstrated that the binocular PRL is not a static anchor but instead shifts systematically with the vergence demand: As the demand for convergence increased, the fixation disparities became increasingly exo (under-convergence), on the order of several arcminutes. At the common starting point of zero vergence demand, however, the subjects in the study by Bowers et al.^21^ did not adopt a uniform direction, as eso- and exo-shifts occurred in roughly equal proportions, and only when convergence was demanded did the distribution become exo-dominant. Our cohort, examined at this zero-demand transition from monocular to binocular viewing, likewise spanned both directions (Fig. 7), with a predominance of eso-shifts (over-convergence). The direction of the binocular shift thus appears to be an inter-individually variable property, consistent with the heterogeneity in shift magnitude and direction seen across our cohort, so that the relative balance of eso- and exo-shifts may differ between cohorts without suggesting a fundamental discrepancy. Beyond this, our paradigm does not itself introduce a vergence–accommodation conflict. By the parallel-beam, optical-infinity design of the bSLO, both the vergence and the accommodative demand were fixed at zero (see Methods). Because the two demands coincide rather than diverge, no conflict of the kind seen in near paradigms, which load vergence while fixing accommodation, was introduced. Moreover, any proximal (instrument) accommodation evoked by a subjective sense of nearness^42^ would have shifted the focal plane of the eye in front of the retina and thereby degraded the sharpness of the retinal image we recorded; a shift of this kind would therefore have been evident to the operator as a loss of image quality during acquisition. These differing directional biases underscore the adaptability of the oculomotor system across viewing contexts; rather than returning the eyes to their monocular fixation position, the system settles at a distinct, reproducible binocular configuration, introducing or at least tolerating objective vergence offsets of several arcminutes.

As proposed by Reiniger et al.,^6^ such offsets could potentially help horizontally expand the binocular field of high-density sampling. A related argument has been advanced by Gibaldi et al.,^43^ who showed that combining the retinal conjugate surfaces of the two eyes (the blur horopter) enlarges the binocular depth of field of sharp vision relative to monocular viewing. However, introducing a standing objective fixation disparity raises the question of how the visual system avoids diplopia. The magnitude of the disparities we observed falls well within the central dimensions of Panum's fusional area, which is typically estimated to be around 10 to 15 arcmin at the fovea.^44^ Neurophysiologically, this tolerance is supported by the broad receptive fields of disparity-tuned neurons in the primary visual cortex (V1), which can accommodate such microscopic misalignments while maintaining binocular single vision.^45^

It remains an open question whether these micro-vergence shifts represent a beneficial adaptation that places fixation in a more favorable region of the photoreceptor mosaic for sampling or, conversely, an oculomotor limitation. If the latter is true, larger objective fixation disparities might strain the limits of Panum's fusional area, potentially leading to poorer stereoscopic performance, consistent with the classical finding that binocular depth discrimination degrades as disparities increase beyond the horopter.^46^ Investigating how the magnitude of these binocular vergence shifts directly correlates with individual stereoacuity thresholds will therefore be a critical next step. Taken together, our observations suggest that each eye adopts a different, reproducible PRL under binocular compared to monocular viewing, a pattern of intraocular shifts consistent with a micro-scale objective fixation disparity at the level of the retina.

One limitation follows from the use of the monocular PRL as the zero reference. While this monocular calibration defines the zero for each eye, whether it accurately estimates the true binocular zero remains, in principle, unknown.^39^ This uncertainty concerns only the absolute location of the zero and hence the absolute eso- or exo-sign for a given individual; it does not affect the relative monocular-to-binocular shift reported here, which is referenced identically in both conditions.

A further limitation is that binocular fusion was not verified perceptually, as no dichoptic or stereoscopic task was administered during imaging. Likewise, stereoacuity was not measured, so the functional consequences of the observed micro-vergence for fine stereopsis remain to be established.

### Motor Strategy and Binocular Coordination of FEMs

Our data point toward a context-dependent shift in oculomotor strategy during binocular vision. Under monocular conditions, microsaccades are thought to repeatedly relocate gaze back to the exact functional anchor.^4^^,^^29^ However, our observation of sustained, stable fixation despite the active introduction of objective fixation disparities suggests a potential reprioritization under binocular viewing.

Our decomposition of the fixational signal lends direct support to this notion of a reprioritized motor strategy. Because microsaccade rates remained unchanged while their amplitudes decreased, the binocular advantage does not appear to emerge from a reduction in the number of microsaccades but rather from a finer-grained control of their magnitude, complemented by a tighter intersaccadic drift. Such a strategy is consistent with a system that, with binocularity, favors small and well-regulated fixational movements that preserve binocular correspondence over larger excursions that might transiently disrupt it.

This behavior highlights the complex binocular coordination of miniature eye movements. Although microsaccades are generally highly conjugate (yoked) between both eyes to maintain overlapping visual fields,^47^^,^^48^ intersaccadic drift contains a much larger unyoked, independent component.^49^ It is plausible that under binocular stimulation, the oculomotor system relies more heavily on the slow vergence-like components of drift to maintain alignment within Panum's area. Rather than triggering conjugate microsaccades that might transiently disrupt the binocular correspondence, the system might tolerate a standing objective fixation disparity.^50^ The bSLO architecture, offering intrinsically synchronized retinal tracking of both eyes, is well suited to further disentangle the binocular coordination of FEMs in future studies.

## Conclusions

Our bSLO measurements provide evidence that binocular viewing alters microscopic oculomotor behavior, enhancing spatial stability through smaller microsaccades and tighter drift while recruiting distinct retinal coordinates. Although the direct link between these motor changes and binocular sensory performance remains to be fully elucidated, these findings highlight the flexibility of the binocular visual system in coordinating motor control at the photoreceptor level.
